# EGFP gene transfection into the synovial joint tissues of rats with rheumatoid arthritis by ultrasound-mediated microbubble destruction

**DOI:** 10.3892/etm.2014.1579

**Published:** 2014-02-24

**Authors:** XIANG-XIANG JING, JIE LIU, BING-ANG YANG, SHAO-QING FU, TANG-NA WU, DONG-LIN WANG

**Affiliations:** 1 Department of Medical Ultrasonics, Hainan Provincial People’s Hospital, Haikou, Hainan 570311, P.R. China; 2 Department of Emergency Orthopedics, Hainan Provincial People’s Hospital, Haikou, Hainan 570311, P.R. China

**Keywords:** ultrasound, microbubble, rheumatoid arthritis, gene transfection

## Abstract

The aim of the present study was to explore the feasibility of enhancing green fluorescent protein (EGFP) gene transfection into the synovial joint tissues of rats with rheumatoid arthritis (RA) by ultrasound-mediated microbubble destruction. An optimal SonoVue dose was determined using 40 normal rats categorized into five groups according to the various doses of microbubbles used. At 1 week after ultrasound irradiation, the rats were sacrificed. Damage to the joint synovial tissues was observed with hematoxylin and eosin histopathological staining under a microscope. A further 44 normal rats were used to establish a rat model of RA, and were then categorized into four groups: EGFP, ultrasound + EGFP, microbubbles + EGFP and ultrasound + microbubbles + EGFP. The last group was irradiated with ultrasound for 10 min following the injection of 300 μl SonoVue and 10 μg EGFP into the joint cavity. Rats were sacrificed after 3 days and synovial tissue was collected from the knee joints for observation of EGFP with fluorescence microscopy and analysis by quantitative polymerase chain reaction. EGFP expression was observed in the synovial tissues of all groups. However, high EGFP expression levels were observed in the ultrasound + microbubbles + EGFP group. No statistically significant differences (P>0.05) were observed in the EGFP expression levels between the EGFP, ultrasound + EGFP and microbubbles + EGFP groups. However, EGFP expression levels in the EGFP, ultrasound + EGFP and microbubbles + EGFP groups significantly differed (P<0.05) from that in the ultrasound + microbubbles + EGFP group. Therefore, ultrasound-mediated microbubble destruction improved EGFP transfection efficiency into the joint synovial tissues of rats with RA.

## Introduction

Gene therapy is a medical procedure in which exogenous genes (target genes) are transferred into target cells (receptors) via certain vectors. The method is typically used to compensate for defective genes or protein secretion in target cells. In addition, gene therapy may be used to express certain receptors on target cells using the protein product of the transgene, resulting in the target cells obtaining new biological behaviors or functions. Therefore, gene therapy may be used in the treatment of a number of diseases (1).

Gene therapy for joint diseases has become increasingly studied in recent years. Rheumatoid arthritis (RA) is the most common systemic autoimmune joint disease, characterized by chronic inflammation, abnormal immune responses and excessive synovial fluid (2). In 1994, the incidence rate of RA worldwide was reported to be 0.3–1% (3). The majority of patients with RA suffer from severe joint pain, which may result in premature retirement from work. This is a problem not only for the patients, but also for their families and society. Therefore, identifying an effective treatment for RA is likely to have a broad clinical and social significance. As the pathogenesis of RA is not fully understood, existing treatment methods are unsatisfactory (4). A number of drugs, including nonsteroidal anti-inflammatory drugs, only treat certain symptoms, for example, by diminishing inflammation or relieving pain. In addition, biological and immunosuppressive agents may produce severe side-effects with long-term use, while only alleviating the symptoms. Stem cell transplantation is able to improve the symptoms of RA, however, it is an expensive procedure that usually results in relapse.

By contrast, gene therapy presents a promising potential for treating the disease more comprehensively. However, RA is not a single gene disorder. RA patients may carry specific susceptibility genes that allow the disease to manifest following exposure to certain external conditions (5). Gene therapy in the treatment of RA may therefore be more comprehensive due to its ability to inhibit or enhance certain aspects of RA pathogenesis (6).

The results of RA gene therapy in laboratory tests and animal experiments have been satisfactory (7–9), which demonstrates the potential for clinical application. However, a number of issues need to be overcome prior to clinical use. Current gene therapy vectors are categorized as viral or nonviral. Viral vectors have poor security and are also immunogenic and mutagenic. A retroviral vector has a small capacity, short survival time and is potentially carcinogenic. Liposomal transfection has low efficiency, poor specificity and short expression time. Thus, the identification of a vector that is safe, highly efficient, target-specific and that can be stably expressed with low immunogenicity is the primary pursuit in the field of gene therapy (10).

Previous studies have shown that microbubble ultrasound contrast agents may be used as targeting carriers of medicinal and genetic material (11–13). Natural bubbles in the liquid alternatively compress and expand with the application of ultrasound, a process known as cavitation. A natural bubble is a type of natural cavitation nucleus. Under the effects of a low sound field, the compression and expansion of natural bubbles become balanced, which is then referred to as stable cavitation. However, under the effects of a high sound field, the balance between the compression and expansion of natural bubbles is lost and the bubbles are broken. This type of cavitation is known as inertial or unstable cavitation. Microbubble ultrasound contrast agents contain various gas components, which may reduce the ultrasonic cavitation threshold and enhance the cavitation effect. The cavitation effect of ultrasound contrast agents on surrounding tissues is currently considered to result mainly from the inertial cavitation of microbubbles (14), whereas stable cavitation is primarily used to provide nonlinear signals required for diagnosis. A large amount of energy is released when microbubble inertial cavitation occurs, affecting nearby materials and resulting in cavitation. This produces various biological effects on cells, capillaries (15) and surrounding tissues (16). Specific studies have shown that ultrasonic irradiation itself promotes *in vitro* and *in vivo* gene transfection, whereas the inertial cavitation induced by the breaking of microbubble ultrasound contrast agents significantly enhances the gene transfection rate (17–22).

A previous study investigated the treatment of RA using medicine-wrapped microbubbles (23). Another study reported that ultrasound-mediated microbubbles are able to transfect plasmid DNA successfully into normal joint synovial tissues (24). However, there have been limited studies concerning the treatment of RA using gene-transported microbubbles mediated by ultrasound. The present study utilized microbubble ultrasound contrast agents with a high sound field to investigate the ability of this technique to improve the transfection efficiency of enhanced green fluorescent protein (EGFP) in the synovial membrane tissue of RA model rats. The aim of the study was to validate the feasibility of using this gene therapy method in the treatment of RA, to provide a novel, more ideal method of RA treatment.

## Materials and methods

### Optimization of the SonoVue dose

For optimization of the dose of SonoVue (sulfur hexafluoride microbubbles; Bracco, Milan, Italy), 40 normal Wistar rats of clean grade were purchased from Dongchuang Laboratory Animal Science and Technology Inc. (Changsha, China). There were 20 male and 20 female rats weighing between 260 and 280 g (average weight, 270.6±6.6 g). The study was performed in strict accordance with the recommendations in the Regulations for the Administration of Affairs Concerning Experimental Animals (Approved by the State Council on October 31, 1988 and promulgated by Decree No. 2 of the State Science and Technology Commission on November 14, 1988). The animal use protocol was reviewed and approved by the Institutional Animal Care and Use Committee of Hainan Provincial People’s Hospital (Haikou, China). The rats were categorized into five groups with eight rats (16 knee joints) in each group: Group 1, ultrasound + 100 μl microbubbles; group 2, ultrasound + 200 μl microbubbles; group 3, ultrasound + 300 μl microbubbles; group 4, ultrasound + 500 μl microbubbles; and group 5, ultrasound irradiation (control group). SonoVue was injected into the articular cavity following anesthetization and disinfection. The articular joint cavity was irradiated continuously by color Doppler ultrasonography (HDI 4000; Philips Healthcare, Andover, MA, USA) at a detector frequency of 10 MHz, mechanical index (MI) of 1.3 and a depth of 2 cm for 10 min. Rats were sacrificed after 1 week and the damage to the joint synovial tissues was examined with hematoxylin and eosin histopathological staining using a microscope (CX31, Olympus, Tokyo, Japan).

### Plasmid extraction and identification

pEGFP-C1 plasmid was donated by Chongqing Medical University Institute of Liver Disease. Plasmids were extracted from *E. coli* using a plasmid DNA purification kit (Qiagen GmbH, Hilden, Germany). The concentration, measured using a spectrophotometer, was 1 mg/ml.

Human gastric cancer cell line SGC-7901 was cultured in a minimum essential medium (MEM) containing 10% newborn calf serum. The culture was incubated in conditions of 5% CO_2_ and 37°C in a thermostatically sealed incubator (relative humidity, 95%) for cell passaging. Cell growth was observed using an inverted microscope. The cells were passaged once every 2–3 days. Cells in a logarithmic growth phase were inoculated into 12-well plates and the cell density was adjusted to 5×10^4^ cells/ml. The samples were incubated and cultured for an additional 24 h. Cells were then cultured until the confluence reached 70–80%. Next, 20 μl plasmid at a concentration of 1 μg/μl was added to serum-free MEM to produce a final volume of 100 μl. EndoFectin transfection reagent (60 μl) was diluted with serum-free MEM to 100 μl, and then mixed with the plasmid at a ratio of 1:3. MEM culture medium (1 ml) containing 10% calf serum was added to each well in a 12-well plate. The plasmid-EndoFectin solution (15 μl; Qiagen, Hilden, Germany) was added to each well while swirling the plate gently. Finally, the 12-well plate was incubated again for continued culturing. The transfection efficiency was observed under a fluorescence microscope 16 h after transfection.

### Establishment of an RA rat model

In total, 50 normal Wistar rats of clean grade (males 25; females, 25; weight, 258–279 g, average weight, 261.57±5.42 g) were purchased from the Dongchuang Laboratory Animal Science and Technology Inc. Inactivated Bacillus Calmette-Guérin vaccine (Beijing Biological Products Institute, Beijing, China) was added to Freund’s complete adjuvant (Sigma-Aldrich, St. Louis, MO, USA) at a concentration of 4 mg/ml and the mixture was stored at 4°C until required. Type II collagen (Sigma-Aldrich) was dissolved into 0.01 mol/l acetic acid to a final concentration of 4 mg/ml and mixed well at 4°C overnight. The type II collagen-acetic acid solution was added dropwise into cold Freund’s complete adjuvant at a 1:1 ratio until completely emulsified. A 0.25 ml aliquot of the emulsion was intradermally injected into the left rear foot, tail roots and back of each rat. After 7 days, a booster injection was administered in the same manner. Rats with significantly swollen limbs with ankle diameters ≥2 mm were considered to be successful models.

### Experimental groups

Successful RA models were set up in 44 RA rats which were categorized into four groups of 11 rats (22 knee joints): Group 1, EGFP plasmid; group 2, ultrasound + EGFP plasmid; group 3, microbubbles + EGFP plasmid; and group 4, ultrasound + microbubbles + EGFP plasmid.

Group 1 rats were injected with 10 μg plasmid into the articular cavity. Group 2 rats were subjected to continuous color Doppler ultrasonography (Philips HDI 4000) at a detector frequency of 10 MHz, MI of 1.3 and depth of 2 cm for 10 min, following the injection of 10 μg plasmid into the articular cavity. For group 3 rats, SonoVue was diluted in 5 ml normal saline solution (0.9% NaCl), 300 μl of which was mixed with 10 μg plasmid. The mixture was placed at 4°C for 30 min and then injected into the articular cavity. Group 4 rats were continuously exposed to color Doppler ultrasound following the injection of diluted SonoVue and 10 μg plasmid into the articular cavity.

### Quantitative polymerase chain reaction (qPCR)

After three days, the synovial tissue of knee joints in each group were removed and partly observed by fluorescence microscopy (BX10; Olympus, Tokyo, Japan) at 480 nm wavelength stimulation. Sections of the tissues were immediately frozen in liquid nitrogen. Total RNA was extracted using an RNA extraction kit (Axgen, Inc., Redwood, CA, USA), whereas mRNA was reverse transcribed into cDNA using a reverse transcription kit (Fermantas, Thermo Fisher Scientific, Waltham, MA, USA). Pre-amplification was performed for each group with cDNA as a template, GAPDH primers as an internal reference (sequences 5′-TCCCTCAACATTGTCAGCAA-3′ and 5′-AGCTCCACAACGGATACATT-3′) and GFP primers (sequences 5′-ACAAGTTCAGCGTGTCCG-3′ and 5′-CTCGTTGGGGTCTTTGCT-3′) as quantitative primers (Guangzhou Funen Gene Company, Guangzhou, China). The length of the target fragment was 714 bp and the amplification products were detected by electrophoresis in 1.5% agarose gel.

Amplification was performed using an Eppendorf quantitative PCR system (Eppendorf, Hamburg, Germany) at the following cycle temperatures and times: 95°C, 10 min; 95°C, 10 min; 55°C, 20 min; 72°C, 15 min; 95°C, 15 min; and 72°C, 15 min for 30 cycles. The amplification and melting curves of qPCR were confirmed following the reaction. Finally,

$$2^{-\Delta \Delta \text{Ct}}=\underset{\text{sample}}{\underset{︸}{2-[({\text{C}}_{\text{target}}-{\text{C}}_{\text{reference}})]}}-\underset{\text{control}}{\underset{︸}{\left[({\text{C}}_{\text{target}}-{\text{C}}_{\text{reference}})\right]}}$$

the expression difference of EGFP in various organs was analyzed by qPCR. The relative quantitative method was used to calculate the ratio between EGFP and the internal reference GAPDH, which represents the relative level of EGFP mRNA. The following formula was used:

### Statistical analysis

Data were processed and analyzed using SAS statistical software, version 9.0 (SAS Institute, Inc., Cary, NC, USA). Measurement information and statistical data are expressed as mean ± SD. Although the data verification revealed a normal distribution, comparisons between groups were performed by variance analysis with the Student-Newman-Keuls test. P<0.05 was considered to indicate a statistically significant difference.

## Results

### SonoVue optimization

**Figure 1:**
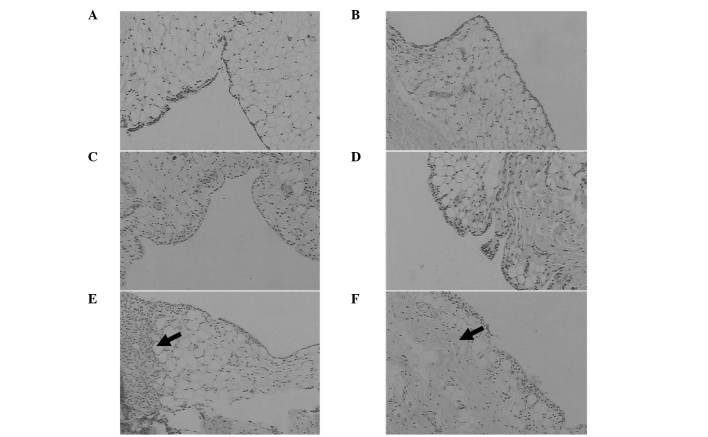
Pathological images (hematoxylin and eosin; magnification, ×40) of (A) control, (B) ultrasound + 100 μl microbubble, (C) ultrasound + 200 μl microbubble, (D) ultrasound + 300 μl microbubble and (E and F) ultrasound + 500 μl microbubble groups. (A–D) Synovial cells are arranged neatly in single or double layers and the synovial membranes surrounding the structures are arranged in neat rows. (B–D) A small amount of dilatation and congestion of blood capillaries is observed. (E) Synovial tissues exhibit hyperplasia and are arranged in a disorderly manner (arrow). (F) Muscle fiber structure is dissolved and fractured (arrow).

No significant damage to synovial tissue was observed in the ultrasound + 100 μl microbubbles, ultrasound + 200 μl microbubbles, ultrasound + 300 μl microbubbles and control groups. However, in the ultrasound + 500 μl microbubbles group, synovial cell swelling was observed. Cell morphology was round, oval or polygonal and synovial tissues were hyperplastic. The cells were disorderly, and capillary proliferation, structural fractures and dissolution of muscle fibers, and inflammatory cell infiltration were identified in the surrounding tissue (Fig. 1).

### Plasmid identification

**Figure 2:**
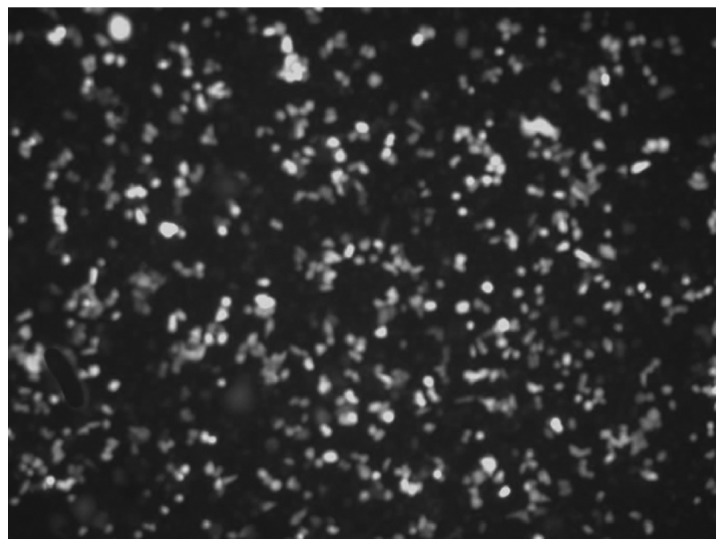
Fluorescence microscope image showing EGFP transfection (magnification, ×100). EGFP, enhanced green fluorescent protein.

The majority of human gastric cancer SGC-7901 cells showed visible green fluorescence following transfection, which indicated that the pEGFP-C1 plasmid was expressed successfully (Fig. 2).

### Rat RA animal model

**Figure 3:**
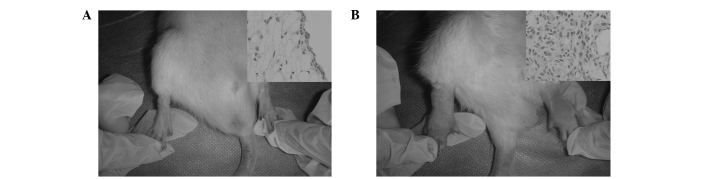
Rat model of RA. (A) Normal rat joints. The pathological slice shows that synovial cells are arranged regularly in a monolayer (hematoxylin and eosin; magnification, ×100). (B) Joints of RA model rats are swollen significantly. Pathological biopsy shows that synovial cells have proliferated significantly and are disorganized. Capillaries are significantly dilated and congested (hematoxylin and eosin, magnification, ×100). RA, rheumatoid arthritus.

The emulsion containing type II collagen was intradermally injected into the left rear foot, tail roots and back of each rat. Booster injections were administered after 7 days in the same manner. After 14 days, all limbs of the injected rats were extremely swollen and ankle diameter was ≥2 mm. Pathological examination of tissue slices confirmed that RA models had been established successfully (Fig. 3).

### Expression of EGFP

**Figure 4:**
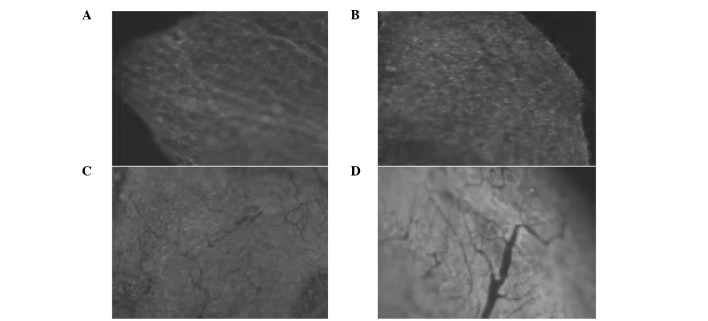
Fluorescence microscopy images showing EGFP expression in rat joint synovial tissues (magnification, ×100) in (A) EGFP plasmid (B) ultrasound + EGFP plasmid (C) microbubble + EGFP plasmid and (D) ultrasound + microbubble + EGFP plasmid groups. EGFP, enhanced green fluorescent protein.

EGFP expression was observed in the joint synovial tissues from all groups. However, particularly high levels of EGFP expression were observed in the ultrasound + microbubbles + EGFP group with fluorescence microscopy (Fig. 4).

**Figure 5:**
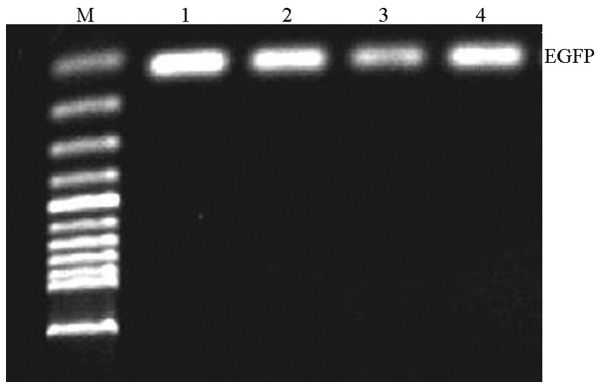
EGFP cDNA electrophoresis results for the synovial tissues of all four experimental groups. M, marker; 1, EGFP plasmid only group; 2, ultrasound + EGFP plasmid group; 3, microbubble + EGFP plasmid group; 4, ultrasound + microbubble EGFP plasmid group; EGFP, enhanced green fluorescent protein.

EGFP cDNA electrophoresis results of the synovial tissues in all experimental groups are shown in Fig. 5. EGFP cDNA was observed in the synovial tissues from all four groups.

### Quantitative analysis of EGFP

**Figure 6:**
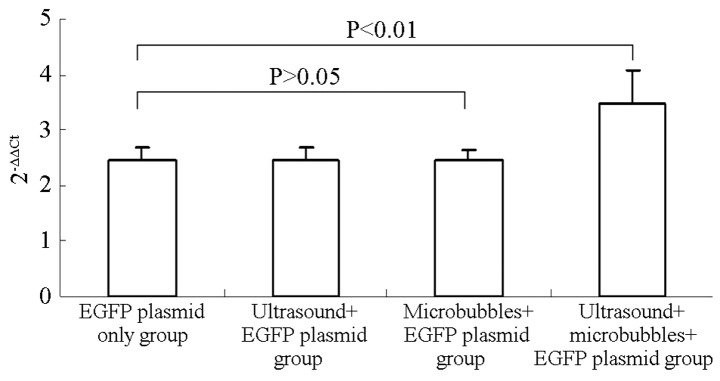
EGFP quantitative analysis results in the synovial tissues of all four experimental groups. EGFP, enhanced green fluorescent protein.

Differences in EGFP plasmid expression were calculated using the relative qPCR 2^−ΔΔCt^ method (ratio of EGFP to GAPDH). The 2^−ΔΔCt^ multiple values of the EGFP plasmid only, ultrasound + EGFP plasmid, microbubbles + EGFP plasmid and ultrasound + microbubbles + EGFP plasmid groups were 2.47±0.19, 2.50±0.17, 2.46±0.19 and 3.47±0.62, respectively. The multiple differences in every group were >1, indicating that EGFP was expressed in each group in varying degrees. No significant difference was observed in the expression level of EGFP between the EGFP plasmid only and the ultrasound + EGFP plasmid or microbubbles + EGFP plasmid groups (P=0.89, 0.93 and 0.82, respectively). However, a significant difference was observed in the expression level of EGFP between the ultrasound + microbubbles + EGFP plasmid group and all the other groups (P<0.01 for all groups) (Fig. 6).

## Discussion

RA is the most common systemic autoimmune joint disease; however, the pathogenesis is not yet fully understood. To date, no satisfactory or effective treatment methods have been introduced. Gene therapy for joint diseases has become increasingly studied in recent years. The synovium is an important tissue in the articular cavity, as the pathological changes in joint disease are closely associated with synovial tissue. The pathological changes of RA begin in the synovium with presentations of synovial hyperemia and edema. Fibrin exudation covers the surface of the synovium and the tissue is gradually invaded by lymphocytes, plasma cells and a small number of multinucleated granulocytes. Eventually, synovial cell proliferation occurs (25). The proliferation of synovial tissue increasingly intensifies and intrudes into the articular cartilage as the disease progresses. Pathological changes, including cartilage damage, extensive intra-articular adhesions and joint space narrowing, eventually occur and cause swelling, pain, apparent limited activity and joint ankylosis and deformity to the affected joint. These changes are also accompanied by bone degeneration and muscle atrophy changes near the joints. Therefore, synovial cells are ideal targets for gene transfection (26).

The success of gene therapy significantly depends on whether the gene delivery system can safely and efficiently transport the target genes into cells and express the genes effectively. Previous studies have shown that microbubble ultrasound contrast agents can be used as targeted carriers of medicinal and genetic material. Ultrasound can be focused in almost all relatively small areas of the body, where it induces the microbubbles to enter the blood circulation and ‘burst’ at designated locations, releasing the carried genes. The oscillations induced by the microbubble burst increase the permeability of eukaryotic cells, promoting the penetration of DNA molecules, as well as improving genetic transduction and expression efficiency (27).

A number of studies have shown that the mechanism of gene transfection mediated by the bursting of microbubbles with ultrasound involves decavitation effects. The decavitation effects of microbubble bursting increase cell membrane permeability. The shock waves and jet flow generated by the bursting of microbubbles function as a driving force to promote the gene combined with the microbubbles to transfer into the target cells and complete gene transfection (24,28,29).

The significance of the injury induced by the cavitation of ultrasound contrast agents varies significantly. For the diagnosis of disease, this injury is adverse and should be avoided to ensure the safety of clinical application. However, when used in disease treatment, including gene transfection and drug delivery, this injury becomes an essential transmission channel. The generation of the appropriate number and size of spillover channels into capillaries and tissue cells should be ensured. By contrast, tissue injury can be minimized to normal levels. Only by this method is it possible to achieve the treatment goals. This method is mainly used for the treatment of cancer where the injury is severe enough to cause irreversible changes to capillaries and surrounding tissues. The parameters affecting the cavitation of ultrasound contrast agents include ultrasonic energy, ultrasonic irradiation time, the amount of contrast agent and the characteristics of microbubbles. In the present study, irradiation was conducted for 10 min under high sound field ultrasound exposure conditions (MI, 1.3), which sufficiently induced microbubble contrast agents to produce inertial cavitation. It was observed that when the dose of microbubble contrast agents was ≤300 μl, the cavitation effect did not cause significant pathological changes in the synovial membrane tissue cells. However, as the dose increased, the effect manifested as small reversible injuries, including specific capillary proliferation in the surrounding synovial membrane tissues and less inflammatory cell infiltration. When the dose of the ultrasound contrast agent increased to a certain level (≥500 μl), the cavitation effect was markedly enhanced, but irreversible injury occurred in the synovial membrane tissues. Therefore, the dose of the contrast agent microbubbles selected for the study was 300 μl.

In the present study, articular cavities were locally injected with contrast agent microbubbles and EGFP transfection was achieved through the direct irradiation of the joint cavities using ultrasound. This method focused the gene therapy at the local intra-articular lesions to ensure that the procedure was safe.

In the study, a rat model of RA was successfully prepared. It was confirmed by biopsy that the synovial tissue of the rats exhibited pathological changes consistent with those of RA. An EGFP plasmid was used in the transfection of the synovial tissues of the RA model rats following the identification of the cell transfection target. Electrophoresis results following the transfection showed that EGFP cDNA was present in the synovial tissues of the rat joints in all four experimental RA groups. However, qPCR analysis showed no significant difference in the EGFP expression levels between the EGFP plasmid only group and each of the ultrasound + EGFP plasmid and microbubbles + EGFP plasmid groups (P>0.05). The EGFP expression level in the synovial tissues of rats with RA in the ultrasound + microbubbles + EGFP plasmid group was statistically significantly higher than those in the other three groups (P<0.05). Thus, the decavitation effect of the microbubbles, generated following ultrasound irradiation, had a pivotal role in the significant improvement of gene transfection efficacy.

In conclusion, this study demonstrated that the efficacy of EGFP transfection into the synovial tissues of rats with RA was improved with the use of ultrasound-mediated microbubble bursting. Therefore, this may be a new approach for the genetic treatment of RA.
